# Addressing the Needs of Transgender Military Veterans: Better Access and More Comprehensive Care

**DOI:** 10.1089/trgh.2016.0040

**Published:** 2017-03-01

**Authors:** Michelle Dietert, Dianne Dentice, Zander Keig

**Affiliations:** ^1^ Texas A&M University—Central Texas, Killeen, Texas.; ^2^ Stephen F. Austin State University, Nacogdoches, Texas.; ^3^ Transgender American Veterans Association, San Diego, California.

**Keywords:** gender identity discrimination, transgender, U.S. military, Veterans Health Administration

## Abstract

**Purpose:** There is a gap in social science literature addressing issues of access and quality of care for transgender military veterans. Psychologists, medical doctors, and other health professionals are beginning to address some of the barriers present in the Department of Veterans Affairs (VA) system that affect veterans who are also transgender and intersex. Over a 7-year period, between 2006 and 2013, 2600 transgender veterans were served by the VA. Data from several surveys revealed that most transgender veterans perceive the Veterans Health Administration (VHA) to be less than accommodating for their special needs. The goal of this study was to investigate the experiences of a sample of transgender veterans with regard to their experiences with healthcare services provided by the VHA.

**Methods:** Using snowball sampling techniques, we were able to recruit 22 transgender military veterans to participate in our study. A combination of telephone interviews and questionnaires provided data from veterans in various branches of the military throughout the United States.

**Results:** Findings indicate that even though the VHA is working to address issues of inequality for transgender veterans, our participants indicated that there are still some problems with administration of care, proper training of staff and physicians, and availability of comprehensive services for the unique healthcare needs of transgender individuals.

**Conclusion:** Since our data were collected, the VA has worked to bridge the gap by focusing on increased training for VHA providers and staff and establishing LGBT programs at VA facilities. However, we suggest that one key area of importance should continue to focus on how mental health and medical providers and ancillary staff are trained to interact with and provide care for their transgender patients.

## Introduction

Although there are only an estimated 1.4 million people in the United States who identify as transgender, current statistics shows they are more likely to serve in the military than their peers.^1,2^ About 48% of Army veterans who served on active duty in Operation Iraqi Freedom (OIF) or Operation Enduring Freedom (OEF) enrolled in and used the Veterans Health Administration (VHA) within 1 year of separation from the military.^1^ The study included >151,000 active-duty members who had deployed and then separated from the Army between 2008 and 2012. Of the >125,000 National Guard and >48,000 Reserve members included in that study, about 57% and 46%, respectively, enrolled with the VHA within 12 months of being demobilized.^3^ These figures illustrate the importance of the VHA to our military veterans.

In 2007, the VHA began focusing on local policies regarding provision of care to transgender veterans and then on June 1, 2011, VHA Directive 2011-024 was issued in an attempt to standardize treatment services for transgender veterans.^4^ The goal of the most updated directive, VHA 2013-003, is to also ensure that adequate healthcare is provided to transgender and intersex veterans. Since transgender individuals are at greater risk of experiencing depression, suicidal behaviors, substance abuse, violence, and homelessness than other people in the population, the authors contend that available evidence suggests the same health risks can be applied to transgender military veterans.^5,6^ Other findings indicate that between 2006 and 2013, the VHA served a total of 2600 transgender veterans.^4^

According to participants in this research study, negotiating their gender identities within the intersection of public/private spaces such as mental health and medical environments is both stressful and complicated. As the data indicate, some of the transgender veterans were faced with a multitude of issues directly related to their gender identity when seeking heathcare with the VHA. Our study adds to the literature dealing with transgender individuals, specifically transgender military veterans who need quality mental health and medical services. As the review of the literature reveals, there is little qualitative research on this subject matter.

Our data focus on the participant's experiences with the Department of Veteran Affairs (VA). It is important to point out that our participants were interviewed during and after the VHA Directive 2013-003 was released by the Department of Veterans Affairs on February 8, 2013. The Directive was written to ensure that adequate healthcare is provided to transgender and intersex veterans. Since our interviews were conducted, the VA has made improvements by providing more training and resources to VHA staff and outreach to transgender veterans. However, at the time of our interviews and currently, the VA does not provide sex reassignment surgery (SRS)^*^ and other procedures needed for transition; an issue for some of the veterans interviewed in our study. Although a step in the right direction, we suggest that the VA continue to focus on training mental health and medical providers and ancillary staff to provide proper care for their transgender patients, including provision of better access to a system that is steeped in bureaucratic rules and regulations relating directly to gender identity. The review of the literature that follows specifically addresses the experiences of transgender veterans with healthcare providers at the VHA as well as some of the current programs that are being utilized to positively impact transgender patient care.

## Review of the Literature

### Transgender military veterans: challenges after service

In a study including 141 trans women veterans, the researchers examined healthcare utilization and potential barriers to accessing care for this population.^7^ They found that the use of the VHA was higher among transgender veterans than the general population of veterans. Approximately 9.3% of the sample sought mental health services from the VHA and the population had higher than average levels of depression when compared to the general nonmilitary population. Veterans in the study also reported suffering from post-traumatic stress disorder (PTSD) along with the need for gender identity counseling. Documented barriers included the cost of health services and the veterans' perceived reaction by medical providers to their gender identity.

One study directly explored discriminatory practices aimed at transgender military personnel.^8^ Focusing on the data collected in 2008 from the University of California, Santa Barbara, the researcher found that in regard to the VA, there were patient care problems and lack of cohesive polices as these relate to transgender veterans. For instance, some patients were given inconsistent information about SRS, were not addressed using pronouns that aligned with their gender identity, and in some cases, were refused service by the VA. Overall, transgender veterans reported that they dealt with many mental and medical health restrictions.

Researchers conducted a study in two VA centers in Oklahoma and Texas in hopes of improving service to LGBT veterans.^9^ The sample consisted of 202 VA providers and 58 LGBT veterans. One finding indicated that 93% (*n*=54) LGBT veterans thought that it was somewhat or very important for the VA to be welcoming, while 81% (*n*=164) of the VA providers shared this same view. Despite these findings, only a minority of both VA providers and LGBT veterans viewed their hospital as welcoming. The researchers concluded that providers should have more training in working with sexual and gender minority clients along with exposure to literature documenting risk factors for LGBT patients.

One study explored the experiences of transgender veterans with the VHA.^10^ The researchers recruited and interviewed 11 transgender veterans from the 2013 Southern Comfort Conference in Atlanta, Georgia. Like the current study, the researchers found that transgender veterans continued to face challenges when seeking healthcare. The VHA not only lacked knowledge in transition-related care but some transgender veterans also experienced insensitivity, harassment, and violence in some VA clinics. Findings also indicated that providers lacked a general knowledge about transgender people.

Regarding clinicians and staff who provide mental health and medical care services to transgender veterans, framers of Directive 2013-003 address the need for more knowledge and education about transgender health issues and specifically state that transgender veterans should be treated with dignity and respect. Professional consultation on complicated cases within Veterans Integrated Service Networks (VISN) or facilities is encouraged by the VA along with cultural awareness and sensitivity education for field staff. For instance, the VHA Transgender SCAN-ECHO (Specialty Care Access Network–Extension of Community Healthcare Outcomes) was introduced in 2011 to train VHA providers on transgender patient care. According to one study, “This program is innovative in its national scope, interdisciplinary team model, and multihub structure.”^11^ Utilizing a 3-year pilot program, the researchers found in the first cohort of learners (*n*=33) that the SCAN-ECHO was very successful in delivering training on transgender care. In fact, respondent's confidence levels to treat transgender veterans increased from 59% to 83% after completing the 14-session, 7-month program. In addition to SCAN-ECHO, the VHA has implemented a 3-year nationwide interdisciplinary e-consultation program on transgender health. The goal of this program is to give consultation to providers who treat transgender veterans at the VHA. In over a 17-month period, 303 e-consults were given to providers on transgender-related care issues to include hormones (*n*=125), primary care concerns (*n*=97), mental health (*n*=63), and psychotherapy (*n*=18).^12^ Overall, e-consultation complements SCAN-ECHO and other trainings offered by the VHA. With regard to total integration of these policies, much rests with the ability of large bureaucratic organizations to minimize barriers and maximize opportunities for advancement of important initiatives such as those discussed above.

## Theory

### The downfall of bureaucratic rational calculation

Max Weber is well known for his sociological perspectives on bureaucracy and its effects on modern-day capitalist societies. Writing in the late 19th and into the early 20th centuries, Weber witnessed the rise of industrialization, with particular attention to institutions reflecting the modern bureaucratic state. Some characteristics of bureaucracy include an explicit division of labor with clear lines of authority, an office hierarchy, written rules and regulations, and management by rules that are emotionally neutral.^13^ The type of legitimate authority found in the bureaucratic state is based on rational–legal domination, where legitimacy is derived from both legal and rational components. The legal component implies “rules or laws which are accepted by subordinates” and the rational component which suggests that “rules are effective and efficient in accomplishing specific and immediate objectives.”^14^ Based on rational–legal domination, an administrative staff carries out tasks and outcomes that are calculated, efficient, and predictable. The end result of bureaucratic, rational calculation is that it fails to sympathize with the human condition in all its forms. Rational action becomes the rule rather than the exception.

The Department of Veteran's Affairs might be described as a ruling organization that “exists insofar as its members are subject to domination by virtue of the established order.”^15^ In other words, the VA is a bureaucracy that functions with strict adherence to rules and regulations. It is part of the military industrial complex that falls under the oversight of highly bureaucratized rational authority figures. Organizations need regulations for order and efficiency; however, the human element or “subjective rights of the individual” are often lost in the bureaucratic shuffle.^15^ Purely rational calculation often results in irrational consequences, particularly for transgender and intersex veterans. For instance, the VHA Directive 2013-003 was passed to address the healthcare needs of transgender and intersex veterans and provides for hormonal therapy, mental healthcare, preoperative evaluation, and medically necessary postoperative and long-term care following SRS. Although a step in the right direction, there are no provisions for SRS services or reconstructive surgery; both important elements in the bigger picture of transgender healthcare.^16,17^

Powers of governance in an organization such as the VA invest authority figures such as doctors and other administrators with a set of rules that are not easily adjusted or changed. These external laws or rules give officials legal–rational authority since there is a demand for expert administration in large institutions such as the VA. Government bureaucrats receive a fixed salary and get jobs based on specific technical skills located within a well-defined hierarchical institution. Rules and official documents are binding and powerful forces against any kind of compromise. In order for the VA to provide the best healthcare for transgender veterans, authority figures at the top levels of the VA hierarchy must re-evaluate the policy limitations set forth by Directive 2013-003. Although programs such as SCAN-ECHO and e-consultation are steps in the right direction, allowing for all transition-related care would be most beneficial for transgender veterans.

Utilizing Weber's theoretical perspective on bureaucracy, the researchers in this study chose to investigate the experiences of transgender veterans impacted by rational–legal domination as set forth by policies that impact their healthcare. Challenging the authority of bureaucratic decision-making, the transgender veterans in this study communicate to the reader their experiences with the VA, including suggestions on how the administration can become more accountable by supporting policies that address transgender care of veterans.

## Data and Methods

A total of 22 transgender veterans were interviewed from August 5, 2012, to July 15, 2013. Participants served in the following military branches: Navy, Air Force, Marines, Army, Army National Guard, and Coast Guard. Each individual was given a pseudonym and any other identifying details were disguised or altered to ensure anonymity. Data collection included phone interviews and questionnaires. Fifteen of the participants stated that they had accessed the VHA within 1 year of the interviews. Five of the participants who submitted questionnaires had not used VHA services during the time of the interviews. Two of the participants did not comment when asked about their experiences with the VHA. Refer to Table 1 for a demographic profile of the participants.

**Table 1.** **Transgender Veteran's Demographic Profile**

Name^a^	Race/ethnicity	Age	Gender identity	Branch	Years of service	Education
Susan	White	57	trans woman	Navy	6	Some college
Robin	White	50	trans woman	Air Force	22	Master's
Emily	White	41	trans woman	Air Force	20	High school diploma
Mary	White	41	trans woman	Air Force	4	Master's
Jenny	White	64	trans woman	Navy	9	BS degree
Holly	White	58	trans woman	Air Force	19	Master's
Amy	White	56	trans woman	Navy	1.5	Some college
Michelle	White	65	trans woman	Marines	3	Master's
Andrew	White	31	trans man	Army National Guard	6	Senior in college
Donna	White	48	trans woman	Navy	25	Master's
Paul	White	56	trans man	Marines	19	Master's
David	Hispanic	46	trans man	Coast Guard	2	Master's
Steven	Unknown	Unknown	trans man	Army	8	Unknown
Pete	American Indian	57	trans man	Army	4	Associate's
Lynn	Biracial	56	trans woman	Marines	3	None
Katherine	White	39	trans woman	Navy	6	Senior in college
Katy	White	44	trans woman	Air Force	20	BA degree
Debbie	White	26	trans woman	Marines	3	Technical degree
Melinda	White	28	trans woman	Army	7	Some college
Julia	White	63	trans woman	Army	26	Associate's
Anna	Unknown	49	female (intersex)	Navy	20	Some college
Brittany	Black	71	trans woman	Air Force	4	Some college

^a^ Each participant was given a pseudonym to protect his or her confidentiality.

Using nonprobability sampling, participants were recruited via purposive and snowball sampling techniques. The lead investigator made contact with a member of Service Members, Partners, Allies for Respect and Tolerance for All (SPART*A), an advocacy organization for lesbian, gay, bisexual, and transgender veterans and active duty service members, their families, and their allies. This individual sent out requests for participants through transgender chat rooms and other online sources. People who were interested then contacted the lead investigator through e-mail to set up a phone interview or to request a questionnaire. One participant (Brittany) elected to do both. Each participant gave his/her consent verbally. The purpose of a verbal versus a signed consent form was to ensure confidentiality and to increase recruitment because of the unwillingness of many transgender individuals to speak out about their military experiences. The use of verbal rather than signed consent was approved by a university institutional review board (IRB).

NVivo, an updated version of the NUD*IST (Non-numerical Unstructured Data*Indexing, Searching, and Theorizing) software program, was utilized to organize the transcribed and questionnaire data. Reading through each interview, the lead investigator used NVivo to organize phrases that corresponded to specific research questions. From this analysis emerged themes that would structure the Findings section of this research project. From the 22 interviews, a total of 12 transcripts were transcribed and uploaded into NVivo. An additional 10 questionnaires were uploaded into NVivo. Examples of research questions include the following: (1) Why did you join the military? (2) Tell me about your experiences as a service member when you were actively serving our country. (3) When you were serving, how did you negotiate your gender identity within the gender expectations of the military? (4) As a transgender veteran, have you been denied healthcare or turned down for any treatment that you needed because of your gender identity? (5) What kind of policy changes do you think could be added or eliminated that impact transgender veterans and those who are currently serving? After the data were organized into themes, a node report was generated providing the researchers with the raw data used to construct the Findings section.

## Findings

Our data focus on the participant's experiences with the Department of Veteran Affairs. Our participants were interviewed during and after the release of VHA Directive 2013-003 by the Department of Veterans Affairs on February 8, 2013. The Directive was written to ensure that adequate healthcare is provided to transgender and intersex veterans. It is important to note that at the time of our interviews, the VA had still not addressed the issue of funding SRS and other surgical procedures; an issue for some of the veterans interviewed in our study. The data reveal that in some cases, there was confusion about what types of healthcare would be covered by the VA at the time of interviews. There were also some concerns about what might happen in the future for transgender veterans who need care at the VA. This uncertainty is a possible reflection of the transitional policies being drafted during the time frame of data collection. The comments that follow focus on transgender care and general healthcare issues.

Transgender individuals, and particularly transgender veterans, are often misunderstood by healthcare providers.^18^ These negative experiences can influence whether or not transgender veterans decide to seek healthcare and continue with ongoing and preventative treatment. The VHA Directive 2013-003 has ensured that VA medical personnel are educated about the needs of the transgender and intersex population.^19^ Brittany (trans woman) discusses her first appointment with the VA:

So I went to the VA for my first appointment with my primary nurse or whatever they call it. Actually I was sitting there in a skirt and stuff, and the lady came out and called (male name deleted). I wasn't about to jump up then. There were two sections so she went to the other section. So when she did that, I stood up. And when she turned back around, I kind of motioned to her. Now she wasn't disrespectful. She just wasn't trained in etiquette I guess. I was trying to find out if there were other transgender people coming through. And at that point, I think I said “Are there other people coming through like me?” And she said “You mean drag queens?” I said “No, I'm not a drag queen.” Other than that, it's been pretty nice… Back to my first visit with the nurse, I asked her if there was anything they could do about electrolysis. She said she would ask.

According to Brittany, she does not believe the nurse was intentionally disrespectful. Had she received a minimum of training, she would have known to ask Brittany how she self-identified her gender. This behavior would align with policies detailed in the VHA Directive 2013-003. Brittany also requested information about electrolysis, a necessary procedure for transgender women that positively contributes to the transition process. Although the VA does provide some healthcare relating to gender identity, electrolysis is currently not part of the healthcare plan for transgender veterans.

Anna (intersex/female) was the only intersex veteran interviewed for this study. She served as a Petty Officer First Class in the Navy. During her service, Anna presented as male, although her gender was often times questioned by others. According to Anna, “Intersex people typically have to deal with being the brunt of jokes because everybody can see that the boy looks more like a girl. The girl acts more like a man.” In other words, many times it is not easy to gender a person as either male or female who has an intersex condition. Anna was laid off from her civilian job and lost her health insurance, so she enrolled at the VA. During her enrollment, she was asked about the name discrepancy on the DD Form 214 (DD214), a document for identifying the veteran's status at discharge. She explained that she underwent SRS and had changed her name. The receptionist in the lobby asked loudly, “Did you go to Thailand to get the sex change?” Humiliated, Anna walked out of the VA and refused to return. She went on to say that she filed a complaint stating the receptionist needed more training to deal effectively with transgender veterans.

Another veteran, Katherine (trans woman), was concerned about getting her name changed in the VA system:

As far as I know I'm listed in the system. I legally had my name changed to Katherine before I was discharged and the VA's records should say only Katherine. I'm sure they've got papers that say otherwise but for the most part my record was gone back through and Kenneth was scratched out with Katherine written right above it.

At the time of our interviews, transgender veterans can ask to have their sex and gender changed in the Computerized Patient Record System (CPRS); however, they must provide a letter from a physician providing specific information as outlined by the Office of Informatics and Analytics.^20^ The veteran must also provide legal documentation to include either a new or amended birth certificate or passport.

Whether identifying as trans women or trans men, most of the veterans in this study agreed that having access to hormone therapy was very important. At this time, the VA does provide cross-sex hormone therapy. Before getting hormone therapy, the individual must be evaluated and monitored over time to ensure positive results. When asked if she had ever been denied healthcare or treatment as it related to her gender identity, Amy (trans woman) had this to say:

No, I have never been turned down from the time that I began my transition. I started with black market hormones until I had found out that the Phoenix VA was providing treatment back in 2005.

Before going to the VA, Steven (trans man) educated himself on the transition process. He wanted to make sure that he was prepared to make informed decisions about his care.

Of course I did my research so I had read, studied, printed and highlighted the standards of care. I put together precisely what was needed for them to give me. So I got my referral to endocrinology. I went to endocrinology with everything that they needed. They didn't even have to interview me. I gave them a written, signed document that they just stick in my file so that they didn't have to bother to go through it. You know outlining what I understood about testosterone and all of that. I was on T within 3 weeks. Within 2 months I had my hysterectomy and within 6 months I had my top surgery done. Right now I'm still busy losing weight and saving money for lower surgery because of course being a vet, SRS is excluded.

As Steven mentioned above, he utilized the “standards of care” as an educational resource on transgender care. The overall goal of World Professional Association for Transgender Health (WPATH) standards of care is to provide clinical guidance for health professionals to assist transsexual, transgender, and gender-nonconforming people with safe and effective ways to achieve overall health, psychological well-being, and self-fulfillment.^21^ For Steven, going into the VA with knowledge about transition helped speed up the process. He stated that he was denied SRS by the VA:

They will take care of everything except SRS. Actually a trans man can go get phalloplasty and then come back to the VA and get their erectile device paid for and installed by the VA because you're then just a dude who can't get it up because your transition is considered complete.

David (trans man) explained a postoperative procedure mentioned by Steven in this way:

So for instance, if one does have genital reconstruction but there is no urethral extension, the urethroplasty that is done so that they can stand and void, the VA will provide that surgery because it's not SRS. It's just male enhancement surgery. They will put in an erection pump because you have erectile dysfunction and it enhances your life as a man. So it's interesting that they are willing to provide certain surgeries but not others. I'd like to see them at least begin to refer people out of network to surgeons who are proficient in those surgeries and cover then partially or fully depending on status of the vet.

As mentioned previously, transgender care as we know it may change in the near future since the VA is currently considering providing SRS to transgender veterans. In addition, since the time of Steven's and David's interviews, more is being offered to transgender individuals who seek to transition. Some U.S. health insurance companies now cover transgender care to include employer provided group plans, Medicare/Medicaid, and some state insurance plans.^22^

Pete (trans man) was diagnosed with severe PTSD. He had this to say when asked about the healthcare he had received from the VA:

They got me some real good therapy going for my PTSD. Now that I've transitioned, they have been very good about that. They won't change your name on your military records or when you were considered female. But like all of my records now are under my male name of Pete. Because of my PTSD, they take care of my…I see a psych doc regularly and they give me my anti-depressant medications. They give me my testosterone which I was shocked that they would do that.

The Department of Defense (DoD) allows name changes in the Defense Enrollment Eligibility Reporting System (DEERS) and on the Military Discharge Record or DD214 by submitting DD Form 149, which is an application for correction of Military Record.^23^ Although it is not guaranteed that the Board of Corrections for Military Records will accept a correction, this procedure is currently the best way to request a name change. At the time of the interview, this option was not available for Pete. Debbie (trans woman) stated that the Veterans Benefits Administration (VBA) was “dragging their feet” to get her on disability.

I was in from 2005 to July 2008. Honorably discharged and all of that. I did my time and got out. I was a paratrooper in the military. I've got a lot of jumps under my belt and I was over in Iraq for 15 months. I did 20 or so jumps. I got blown up like three times or something. So I'm not going in to scam the system or any type of thing. And I know a lot of people do but I have a harder time turning my neck. I can't touch my chin to my shoulder like not even close. I can't touch my chin to my chest. I've lost a lot of motion in my neck. I have the ringing in the ears and went in for PTSD also. I have a lot of issues I guess. So I'm just trying to work out a lot of things and seeing what happens. So it's just one of those things where I'm fighting them and they are just dragging their feet about it.

As David stated, regardless of transgender status, it can “take months to years” to get on disability. At this time, the backlog in disability claims continues to be addressed by the VA in an effort to provide care for all veterans.

What these experiences illustrate is that regardless of gender identity, transgender veterans need access to healthcare. As Brittany (trans woman) mentioned in her interview regarding the DoD:

Well you know one of the lies that they told us when we went in back in 1960 was you serve us for 4 years and we'll take care of you for the rest of your life. And that has been not a complete truth.

It is evident that more needs to be done to ensure that our transgender and intersex veterans are provided adequate healthcare by the VA that includes SRS and other surgeries needed to complete the transition process. As David (trans man) mentioned above, “it's interesting that they are willing to provide certain surgeries but not others.”

Another trend found in the data is that some transgender veterans either had not enrolled or chose not to seek healthcare at the VA. For Julia (trans woman), it was just a matter of taking the time to enroll. When asked if she used the VA, she had this to say:

No in fact I keep saying that I need to go register at the VA because I know they need the body count to get their funding, to keep their funding. But I just haven't registered at the VA so…And my husband is 11 years older than me and he has not registered at the VA either so we need to both go there.

Katy (trans woman), on the contrary, felt apprehensive about the VA. However, because hormonal therapy was becoming too much of a financial burden, she finally made an appointment to enroll:

I haven't been brave enough to venture into that yet but I think I'm getting close. I've got an appointment on Friday so we will see. Paying for hormones out of pocket is getting very expensive and I think I'm going to see what the VA can do for me.

Donna (trans woman) stated “I've never sought treatment at the VA related to my gender identity” while Paul (trans man) said “I don't have anything to do with the veteran's administration and that's my choice.” Michelle (trans woman) stated that she did not use the VA because “I do not trust them.” Jenny (trans woman) chose to use her own private physician rather than use the VA for her healthcare:

I don't use the VA healthcare system. All my private physicians know about my transition and I've never had anything but positive reactions with no change in my healthcare quality.

What this dialogue illustrates is that some transgender veterans are not getting all of the treatment that they need and they do not appear to have full confidence that the VA is a reliable source of healthcare. There is a need for more community-based research that brings together the transgender community and VA staff so as to “diminish prejudice, reduce stigmatization, and encourage transgender veterans to obtain needed healthcare.”^18^ Programs such as SCAN-ECHO and e-consultation are just two ways that the VA is educating providers about transgender care. However, some of the comments made by our participants illustrate the necessity of ensuring that programs such as these continue to be developed to provide a comprehensive approach to transgender care needs within the VA.

## Discussion

When it comes to the issue of gender identity, the question remains as to why the VA provides some treatments for transgender veterans while excluding others. Whether or not transgender people should be allowed to have SRS and other transition-related procedures has been questioned not just by the VA but began back in the 1960s when Johns Hopkins became the first academic institution to perform SRS.^24^ As some of our participants illustrate, getting the transgender care can still be a struggle for them. When asked what policy changes should be added and/or eliminated, our participants made several recommendations. Amy (trans woman) suggested that full treatment, to include SRS, would help to reduce anxiety.

I think it would behoove them (the VA) to provide for reassignment surgery because the cost would outweigh the costs of treating the individual for a number of suicide attempts and mental health issues.

Robin (trans woman) commented that “retired personnel should have insurance coverage and access to the full spectrum of transgender healthcare” through Tricare while Jenny (trans woman) mentioned that it was important to “allow transgender vets the same access and rights to services as all other vets.” These recommendations indicate that many of our participants were concerned that all treatments to include SRS be made available to transgender veterans. Otherwise, inadequate healthcare will continue to be an issue.

Transfriendly doctors and staff who can address an array of medical issues were important for participants in this study. Some of our respondents elected not to utilize the VA for their healthcare needs and others have been less than satisfied with the care they have received in that system. The general consensus among the people in our study was that medical practitioners who are familiar with and open to working with transgender people is a key factor in seeking out treatment from the VA or other healthcare institutions. When a transgender patient sees a doctor who is not informed or does not care about their unique needs, improper treatment may result in ongoing health consequences. For military veterans, unequal medical treatment is not only unethical but it also amounts to a broken promise to people who served their country.

The average age of participants in our study was 50. Their collective years of service were 237 and most had at least some college education. There was one intersex respondent, 16 trans women, and 5 trans men. Although the sample is too small to generalize to the broader transgender veteran population, the comments we received indicate that the VA should be an important health resource for anyone who has served in the military. Even though not all transgender veterans in our sample utilize the VA for healthcare, the data we collected reflect many of the same concerns relative to comprehensive and inclusive medical care for transgender veterans in other similar studies.^10^

In ongoing attempts to address concerns of transgender and intersex veterans, the VHA published Directive 2013-003 to establish policy regarding delivery of healthcare to transgender and intersex veterans enrolled in the VA healthcare system. Although this was a step in the right direction, more must be done to ensure that veterans get the healthcare they need. Along with denying SRS, the VA should also consider other treatments necessary to complete the transition process such as electrolysis and reconstructive surgery.

The limitations of the study are attributed to the use of purposive and snowball sampling techniques, which do not require random sampling; therefore, the findings cannot be generalized to all transgender veterans. More specifically, this sample of transgender veterans is not representative of the transgender population in terms of race and ethnicity, class, disability, and age due to the small sample size. Since the time of our interviews, another important limitation that should be mentioned is that our participants were interviewed from August 5, 2012, to July 15, 2013. Although VHA Directive 2011-024 was in place when some of the interviews were conducted, the new VHA Directive 2013-003 was released by the Department of Veterans Affairs on February 8, 2013, to ensure that adequate healthcare is provided to transgender veterans. Therefore, the data reflect a time before and shortly after the 2013 directive was released. Since the time of our interviews, more has been done by the VHA to improve healthcare services for transgender veterans.^25^

However, as mentioned above, there are still no provisions for SRS services or reconstructive surgery; both important elements in the bigger picture of transgender healthcare. This may change in the near future since the Department of Veterans Affairs is currently considering providing SRS to transgender veterans.^26^ If approved, this new rule would replace VHA Directive 2013-003, alleviating some of the issues faced by many of the participants in our study.

## Conclusion

Medical doctors and other healthcare providers agree that transgender individuals who receive the full spectrum of services experience better quality of life than those who do not.^16^ Even though Directive 2013-003 does not currently support the full spectrum of transgender care for those seeking SRS and other surgical treatments necessary to complete transition, the VA has made some significant headway in recent years in treatment protocols and policies for transgender patients. For instance, there is now Veterans Care Coordinators located at VA facilities that support LGBT programs. The Department of Veterans Affairs lists the websites of 28 VA facilities with LGBT programs in several states.^27^ Providing such programs ensures that LGBT veterans have a supportive and safe environment when seeking the care that they need. Two additional VA clinics that specialize in transgender care have also recently opened in Cleveland, Ohio, and Tucson, Arizona. Like the larger clinics and medical centers in the VHA system, these clinics do not provide SRS and other surgical treatments related to transition. Therefore, bureaucratic powers of governance must continue to re-evaluate policy limitations set forth by Directive 2013-003. While the clinic in Tucson serves >100 transgender veterans, some VA hospitals continue to turn away transgender veterans. Policy changes can and will result in better healthcare along with incentives for transgender veterans to use VA facilities.^28^

The VA also provides trainings to VHA staff regarding LGBT healthcare.^29^ As mentioned earlier in this article, SCAN-ECHO and e-consultation have been very successful in delivering training on transgender care to practitioners. The VA also focuses on outreach to LGBT veterans, which is also a step in the right direction.^30^ At this time, the Department of Veterans Affairs is considering providing SRS to transgender veterans. This new “rule” would remove barriers to transition-related care. The review process could take up to 22 months.^26^ If approved, this new rule would replace VHA Directive 2013-003, alleviating some of the issues faced by many of the participants in our study.

With regard to further research, as military organizations begin to re-evaluate their policies, more transgender service members will come out of the shadows. This affords an opportunity for increased dialogue with researchers about personal experiences with the VA and the military in general. Considering the time frame in which our data were collected, more has been done to improve VHA relationships with LGBT veterans. The VHA has made and continues to make system changes that advance LGBT inclusiveness and clinical competence.^31^ However, we suggest that one key area of importance should continue to focus on how mental health and medical providers and ancillary staff are trained to interact with and provide care for their transgender patients. As our participant Donna (trans woman) so eloquently states, the VHA “needs to have clear cut policies to ensure that trans veterans are receiving fair treatment and are being treated with dignity and respect.” With 84% (96 of 114) of all VHA clinics achieving Leadership Status in the 2016 Human Rights Campaign's Healthcare Equality Index (HEI), perhaps the end of bureaucratic, rational calculation is in sight.^31^ As our data illustrate, transgender veterans have a great deal of knowledge and experiences to share with the VHA that can contribute to shaping current and future polices as these relate to their care. Whenever change occurs, especially in large bureaucratic organizations such as the U.S. military, it is particularly important for social researchers to continue collecting and recording empirical data in an effort to ensure that progress continues and does not stall.

## Abbreviations Used

CPRS: Computerized Patient Record System
DEERS: Defense Enrollment Eligibility Reporting System
DoD: Department of Defense
HEI: Healthcare Equality Index
IRB: institutional review board
OEF: Operation Enduring Freedom
OIF: Operation Iraqi Freedom
PTSD: post-traumatic stress disorder
SCAN-ECHO: Specialty Care Access Network–Extension of Community Healthcare Outcomes
SPART*A: Service Members, Partners, Allies for Respect and Tolerance for All
SRS: sex reassignment surgery
VA: Veterans Affairs
VBA: Veterans Benefits Administration
VHA: Veterans Health Administration
VISN: Veterans Integrated Service Networks
WPATH: World Professional Association for Transgender Health

## Notes

^*^ SRS is also known as gender confirmation surgery. In this article, we will use the first term for consistency. Both are terms used by some medical professionals to refer to surgical options that alter a person's physical attributes to match their gender identity.
